# The Development of a Smart Reminder System to Promote Adherence to Technology-Based Intervention and Assessment

**DOI:** 10.1093/geroni/igab046.2115

**Published:** 2021-12-17

**Authors:** Walter Boot, Neil Charness

## Abstract

The overarching aim of the National Institute on Aging funded Adherence Promotion with Person-centered Technology (APPT) Project is to promote adherence to technology-based solutions designed to enhance the early detection and treatment of cognitive decline. The goal is to build and evaluate adaptive, tailored, and integrated technology-based adherence support systems for mobile software platforms that assess and train cognitive skill. The symposium describes the various steps of the development process of the APPT smart adherence support system. N. Charness will present an overview of the APPT project, its aims, and the clinical trials designed to assess the effectiveness of the APPT smart reminder system compared to typical reminder systems. S. Chakraborty will present detailed analyses of past cognitive intervention data to inform understanding of who is likely at risk for poor adherence and how adherence lapses might be predicted in advance to provide just-in-time adherence support. D. Carr will present an exploration of motivating factors for participants to engage in research, and these motivations will be tapped to help develop motivational messages for the APPT adherence support system to be used in the two planned clinical trials. M. Dieciuc will provide additional insights into motivations for engaging in home-based cognitive assessment and training derived from a focus group study. Finally, S. Zhang will describe the results of an initial pilot study examining the effectiveness of motivational reminder messages that match vs. mismatch participants’ own motivations. All results inform the design of the APPT system to maximize adherence.

